# Rare and Unique Case of Midline Anterior Arch of Atlas Non-formation Mimicking Jefferson Type 1 Fracture

**DOI:** 10.7759/cureus.6850

**Published:** 2020-02-03

**Authors:** Srijit Das, Kamalanathan Palaniandy, Azizi Abu Bakar, Zamzuri Idris, Jafri M Abdullah

**Affiliations:** 1 Anatomy, Universiti Kebangsaan Malaysia Medical Centre, Kuala Lumpur, MYS; 2 Neurosurgery, Fakulti Perubatan, Universiti Kebangsaan Malaysia, Kuala Lumpur, MYS; 3 Neurosurgery, Universiti Kebangsaan Malaysia Medical Center, Kuala Lumpur, MYS; 4 Neurosurgery, Universiti Sains Malaysia Health Campus, Kota Bharu, MYS

**Keywords:** atlas, cervical, fracture, paediatric, spine

## Abstract

Cervical spine injuries are rare occurrences in children, especially the congenital anomalies of the atlas vertebra. Any injury involving the craniovertebral junction such as Jefferson fracture, is a valid cause for alarm due to the complex nature of the craniovertebral junction and the morbidity associated with it. We report the case of a 10-year-old male, who had failure of fusion of anterior arch of atlas due to the failure of formation of the anterior midline synchondrosis, and this mimicked a Jefferson fracture. If it was not for the peculiar absence of any corresponding evidence to suggest spinal injury, we might have mistaken this extremely rare but benign anomaly for a Jefferson fracture and subjected the patient to needless surgical treatment. Hence, it is concluded that keen clinical acumen and clear understanding of the developmental anatomy of these patients may be necessary to adequately manage them.

## Introduction

Cervical spine injuries in the paediatric age group are exceedingly rare. The reported incidence of 1.0-1.3% is far less than half of that of the adult population [1]. However, the sequelae in children are more disastrous with reported mortality to be around 40-50% and 60% of those suffering from permanent neurological deficits [1,2]. Hence, the presence of cervical spine injuries in children may raise alarm and place the treating team in duress, more so if it involves the complex and challenging craniovertebral junction which has an even higher morbidity and mortality rate. We report a unique case where the anterior arch of atlas failed to fuse due to the failure of formation of the anterior midline synchondrosis, and this mimicked a Jefferson fracture. To the best of our knowledge, it is the first such case to be reported in scientific literature.

## Case presentation

A 10-year-old male was found in an unconscious state after being knocked down by a car while crossing the road. He had regained full consciousness by the time he was brought to the hospital. He complained of pain over his head and neck, and a paediatric cervical hard collar was applied. Primary and secondary surveys were done; a scalp laceration over the right parietal area was duly sutured. No other injuries were noted.

**Figure 1:**
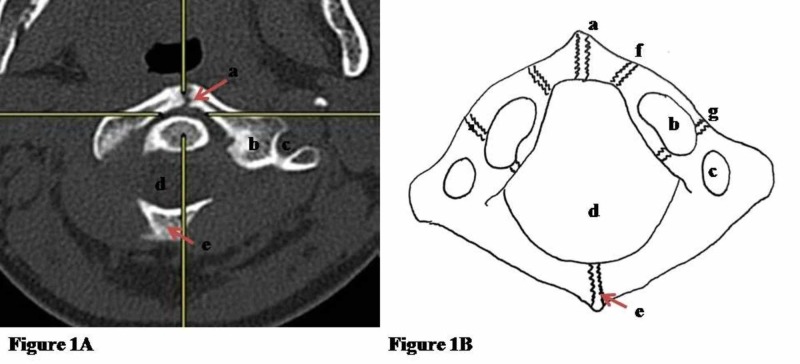
CT scan (axial view) and schematic diagram (superior view) of the atlas. (A) CT scan (axial view) of the atlas showing: a - anterior midline synchondrosis (unformed); b - superior articular facet; c - foramen transversarium; d - spinal canal; e - posterior midline synchondrosis (fused). (B) Schematic diagram (superior view) of the atlas showing: a - anterior midline synchondrosis; b - superior articular facet; c - foramen transversarium; d - spinal canal; e - posterior midline synchondrosis; f - accessory midline synchondrosis; g - neurocentral synchondrosis.

The CT brain was normal. However, his cervical CT showed discontinuity of anterior arch of atlas (Figure 1A).

The initial impression was a rare sub type of Jefferson Type 1 fracture, whereby only the anterior arch of atlas is fractured [3]. However, upon reviewing the CT scan findings, the fracture line was noted to be smooth with presence of sclerosis, as opposed to a fracture line which would be slightly jagged with sclerosis, only a late feature which is associated with the healing process. Furthermore, the patient subsequently was pain free and had full range of motion of his neck without any pain or neurological symptoms. The patient’s diagnosis was then revised as non-fusion of anterior arch of atlas with failure of anterior midline synchondrosis formation. Following clinical confirmation of spinal stability, the cervical collar was removed and the patient was discharged home well after being observed for possible signs and symptoms of neurological deterioration.

## Discussion

Management of children is more complex and cannot be equated as mere management of miniature adults. The constantly evolving biomechanical and physiological conditions are unique to the individual at that given point of time and cannot be replicated in entirety. This makes managing their cervical spine injuries a truly unique and tailored experience. It is a well-established fact that there is a higher incidence of upper cervical spine injury (C3 or higher) in patients younger than eight years old, which is best explained by the anatomical and biomechanical aspects of the developing spine, whereby the fulcrum of movement for the cervical spine is located at the C2-C3 level in younger children, compared to C5-C6 in adolescents and adults [2]. Interestingly, in our patient, who being an adolescent, the propensity for injury should have been the lower cervical spine.

The first two cervical vertebrae are unique in their development. C1, or the atlas, is formed by six primary ossification sites or synchondrosis which may or may not be present in all cases: anterior midline, posterior midline as well as the paired accessory midline and neurocentral synchondroses (Figure 1B) [4].

Ossification of the atlas begins in the seventh week of embryonic period with the initial ossifications of the lateral masses which extends dorsally. The neural arches fuse by the age of four years [5,6]. The anterior arch of the atlas is cartilaginous at birth and forms ventral extension from the lateral masses with the fusion taking place at around 6-8 years [5,7]. The anterior tubercle in the anterior arch gives attachment to the anterior longitudinal ligament and the longus coli muscle while the posterior part bears the smooth oval facet for the articulation of the odontoid process of the second cervical vertebra.

Failure of complete ossification of vertebrae is a well characterized anatomical variation, especially so the atlas which has more ossification sites which increases the probability of such variations. Midline anterior ossification centre has been reported to be often absent in the atlas, thus the anterior arch is often formed by the continuation of the neurocentral synchondrosis. This can be asymmetrical due to various conditions; however the complete fusion of C1 should be complete at around adolescence [4].

In a retrospective study of a large number of patients who underwent CT scan of the cervical spine for various reasons, approximately 1.5 to 5% of the patients had posterior arch of atlas defects, whereas approximately 0.46% had a combination of both anterior and posterior defects and the prevalence of an isolated anterior arch of atlas defect was 0.03% [8]. We extrapolate the true incidence of isolated anterior arch of atlas defects in the population to be rarer than this. The sample population was of those who underwent cervical CT following clinical suspicion of pathologies and not a population-wide prevalence study.

Given the rarity of isolated anterior arch of atlas defects, absence of ossification over the midline of the anterior arch of C1 in an adolescent presenting with trauma may quickly be misdiagnosed as a Type 1 Jefferson fracture [3].

The equally rare occurrence of isolated anterior arch of atlas fractures in children inherent morbidity is associated with injuries of the upper cervical vertebrae. This may result in an injudicious cascade of measures being taken given the highly challenging medico legal scenario pertaining spinal surgery. This undue process itself may cause more harm than benefit to the patient. The key giveaway feature here would be the presence of sclerosis at the site of the defect; it will be further supported by the absence of associated soft tissue injuries.

Managing condition involving the atlas is challenging due to the complex anatomy of the craniovertebral junction. However in the paediatric age group, this is even more challenging due to the presence of various synchodroses coupled with the varying times of ossification and fusion. The intermediate temporality of adolescence further complicates the situation, as most surgeons would find it more convenient to manage them as adults.

## Conclusions

We were fortunate to have avoided unnecessary procedures to this patient due to the suspicion which arose from the sclerotic margin at the site of defect. Therefore good understanding of the developmental anatomy of the atlas prevents such issues and avoids both over and under treatment in this special group of patients.

## References

[1] McAllister AS, Nagaraj U, Radhakrishnan R (2019). Emergent imaging of pediatric cervical spine trauma. RadioGraphics.

[2] Egloff AM, Kadom N, Vezina G, Bulas D (2009). Pediatric cervical spine trauma imaging: a practical approach. Pediatr Radiol.

[3] Jefferson G (1919). Fracture of the atlas vertebra. Report of four cases, and a review of those previously recorded. BJS.

[4] Piatt JH Jr, Grissom LE (2011). Developmental anatomy of the atlas and axis in childhood by computed tomography. J Neurosurg Pediatr.

[5] Truex RC Jr, Johnson CH (1978). Congenital anomalies of the upper cervical spine. Orthop Clin North Am.

[6] Bailey DK (1952). The normal cervical spine in infants and children. Radiology.

[7] Gehweiler JA Jr, Daffner RH, Roberts L Jr (1983). Malformations of the atlas vertebra simulating the Jefferson fracture. AJR Am J Roentgenol.

[8] Hyun G, Allam E, Sander P, Hasiak C, Zhou Y (2018). The prevalence of congenital C1 arch anomalies. Eur Spine J.

